# Loneliness During COVID-19 and Modes of Social Contact Use Among Older Adults

**DOI:** 10.1093/geroni/igab046.2748

**Published:** 2021-12-17

**Authors:** Usha Dhakal, Athena Koumoutzis

**Affiliations:** Miami University, Oxford, Ohio, United States

## Abstract

Due to social distancing and isolation recommendations, COVID-19 resulted in older adults’ greater reliance on technology to contact friends and families. While the mental health of older adults during COVID-19 has been well explored, less is known about how changes in modes and frequency of social contact is associated with loneliness. Using the National Health and Aging Trends Study COVID-19 data, this study assessed how the frequency of varying modes of contact (e.g., phone/email/text, in-person visits, videocalls) during the pandemic was associated with feelings of loneliness during COVID-19 among community-dwelling Medicare beneficiaries (n = 2149). Participants were asked if they felt lonely "more often," "less often," or "about the same" compared to before the outbreak started. Multinomial regression analyses indicated that, compared to those who reported daily in-person visits, the odds of having more feelings of loneliness as compared to about the same as pre-COVID-19 was significantly higher among those who reported having in-person visits a few times (OR=2.17,CI=1.08-4.36), at least once (OR=2.37,CI=1.11-5.04), and never/less than once a week (OR=3.37, CI=1.59-7.16) while controlling for demographics, household, and social network size. Compared to daily use, use of phone/email/text at least once (OR=0.44, CI=0.201-0.965) or a few times (OR=0.76,CI=0.58-0.99) a week was associated with lower odds of reporting more feelings of loneliness versus about the same. Results suggest that greater use of technology that promote social engagement improves social connectedness and decreases COVID-19 related loneliness among older adults, and highlights the importance of older adults’ access to technology, including reliable internet.

